# The complete chloroplast genome sequence of *Acer cinnamomifolium* (Aceraceae), a plant species endemic to China

**DOI:** 10.1080/23802359.2019.1674211

**Published:** 2019-10-09

**Authors:** Minghui Chen, Huijuan Zhang, Ming Jiang

**Affiliations:** Zhejiang Provincial Key Laboratory of Plant Evolutionary and Conservation, College of Life Science, Taizhou University, Jiaojiang, Zhejiang, China

**Keywords:** *Acer cinnamomifolium*, chloroplast genome, phylogenetic analysis

## Abstract

*Acer cinnamomifolium* (Aceraceae) is a plant species endemic to China. In our present study, the complete chloroplast (cp) genome sequence of *Acer cinnamomifolium* was assembled from Illumina pair-end sequencing data. The complete cp genome sequence of *A. cinnamomifolium* was 156,227 bp in size. Totally, 139 genes were identified, including 88 protein-coding genes, 40 transfer RNAs, 8 ribosomal RNA genes, and 3 pseudogenes (*infA*, *ycf1*, and *rps2*). Phylogenetic analysis results showed that *A. cinnamomifolium* is sister to *Acer sino-oblongum*, with a support rate of 100%.

*Acer cinnamomifolium* is an evergreen tree species endemic to China. It is mainly distributed in Zhejiang, Fujiang, Jiangxi, Hubei, Hunan, Guangdong, and Guangxi province. It features grey to blackish-grey bark, leathery leaves, yellowish-green petals, and brownish-yellow fruit. *Acer cinnamomifolium* mainly grows in broadleaf forests at altitudes of 300–1200 m and this tree species can be used as a promising ornamental garden plant. The chloroplast (cp) genome of *A. cinnamomifolium* has not been reported. In this study, we assembled the complete cp genome of *A. cinnamomifolium* and generated a phylogenetic tree to understand its relationship with other *Acer* species.

Leaves were collected at an altitude of 811 m in Yandangshan mountain (28°21′47′′N, 121°03′11′′E), Yueqing City, Wenzhou, Zhejiang province, China. The sample was sealed in a plastic bag and taken to the laboratory. The voucher (CHS2017085) is stored at the Molecular Biology Laboratory at Taizhou University. Leaf DNA was extracted following Doyle and Doyle (1987). A 150 bp paired-end DNA library was constructed, and it was sequenced on an Illumina Hiseq X Ten platform (Illumina, San Diego, CA). A total of 7.2 Gb raw reads were obtained, and the clean reads were *de novo* assembled by NOVOPlasty (Dierckxsens et al. 2017).

The plastome sequence was annotated using Dual Organellar GenoMe Annotator (DOGMA), tRNAscan-SE, and ARAGORN (Lowe and Eddy 1997; Laslett and Canback, 2004; Wyman et al. 2004). The whole length of *A. cinnamomifolium* cp genome (GenBank accession: MN414240) is 156,227 bp, and its overall GC content is 37.9%. The genome is comprised of two inverted repeats (IRs), a large single copy (LSC), and a small single copy (SSC), and their sizes are 26,079, 85,928, and 18,121 bp, respectively. There are 89 protein-coding genes, 40 tRNAs, 8 rRNAs, and 3 pseudogenes in the plastome genome. Most genes occur as single-copy, while 22 genes contain two copies, these include *ndhB*, *orf42*, *rpl2*, *rpl32*, *rps7*, *rps12*, *rrn4.5*, *rrn5*, *rrn16*, *rrn23*, *trnA-UGC*, *trnI-CAU*, *trnI-GAU*, *trnL-CAA*, *trnM-CAU*, *trnN-GUU*, *trnR-ACG*, *trnT-GGU*, *trnV-GAC*, *ycf1*, *ycf2*, *and ycf15*. Three genes, *infA*, *ycf1*, *and rps2*, are proved to be pseudogenes.

A maximum-likelihood (ML) phylogenetic tree including *A. cinnamomifolium* and other 12 *Acer* species was generated using PhyML 3.1 (Guindon et al. 2010), with *Dipteronia sinensis* (Aceraceae) as the outgroup. The *Acer* plant species for ML tree construction were *Acer laevigatum*, *Acer palmatum*, *Acer wilsonii*, *Acer buergerianum*, *Acer truncatum*, *Acer miaotaiense*, *Acer catalpifolium*, *Acer davidii*, *Acer morrisonense*, *Acer griseum*, and *Acer sino-oblongum*. Our results indicated that *A. cinnamomifolium* grouped with *A. sino-oblongum*, and the bootstrap support value was 100%, revealing their close phylogenetic relationship (Figure 1).

**Figure 1.**
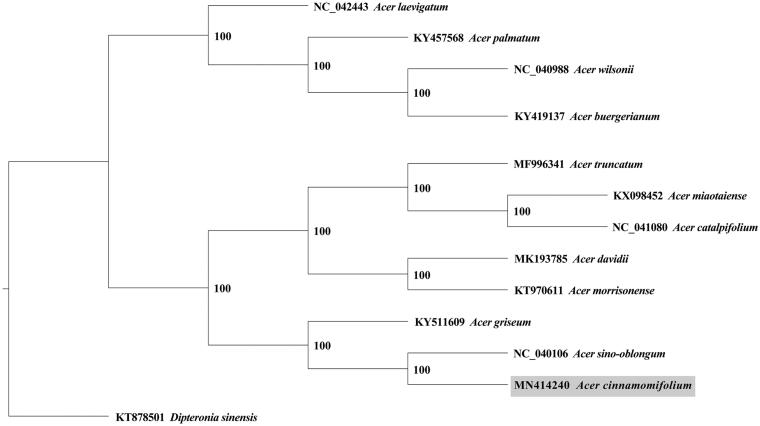
The maximum-likelihood tree generated using the complete chloroplast genome sequences of *Acer cinnamomifolium* and other 12 *Acer plants*, with *Dipteronia sinensis* (Aceraceae) as the outgroup. The numbers next to nodes are bootstrap support values.
